# Supplementary figures and images for: The three NADH dehydrogenases of Pseudomonas aeruginosa: Their roles in energy metabolism and links to virulence
Source: PLoS One. 2021 Feb 3;16(2):e0244142. doi: 10.1371/journal.pone.0244142 (PMC7857637; doi:10.1371/journal.pone.0244142)

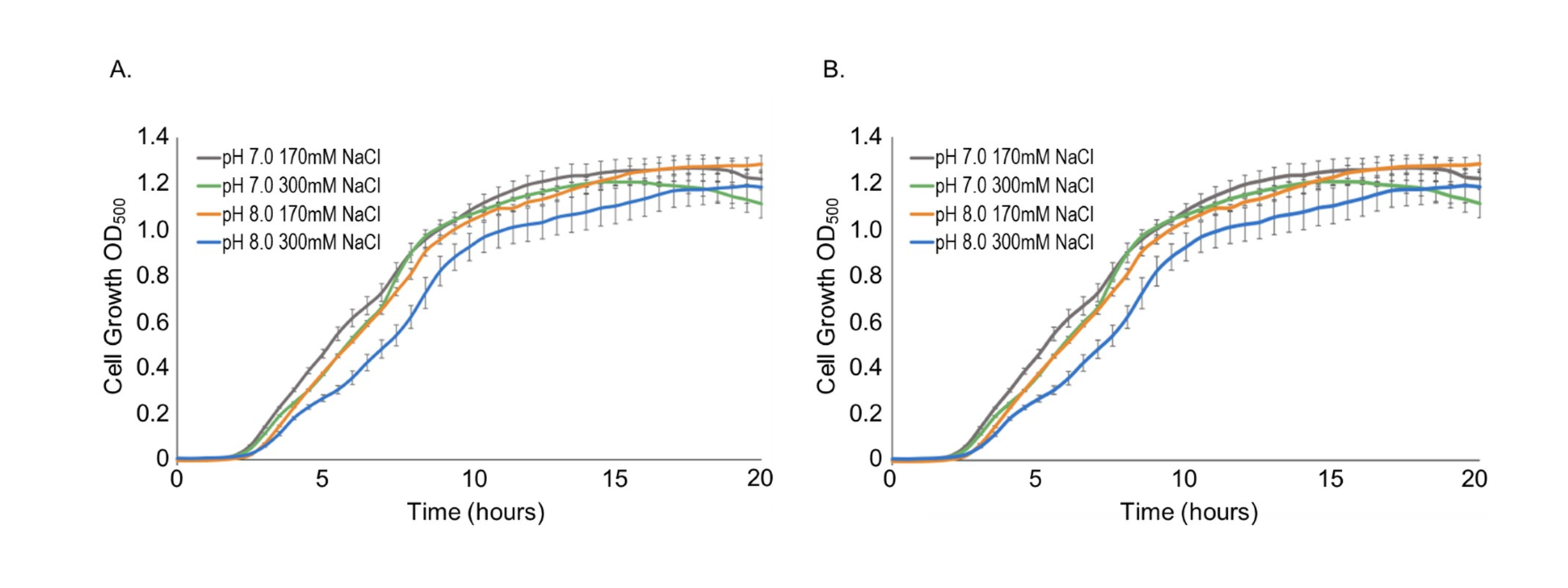

Supplement: S1 Fig — Growth curves of wild type PAO1 in LB (A) and SCFM (B). Changes in OD500 were measured using a Tecan Infinite M1000 Pro plate reader during 20 hours of growth at 37°C with continuous orbital shaking at 217 rpm. Each curve was constructed using two biological replicates with three technical replicates each, with standard deviation calculated accordingly and represented as error bars. (TIF) [file pone.0244142.s001.tif]

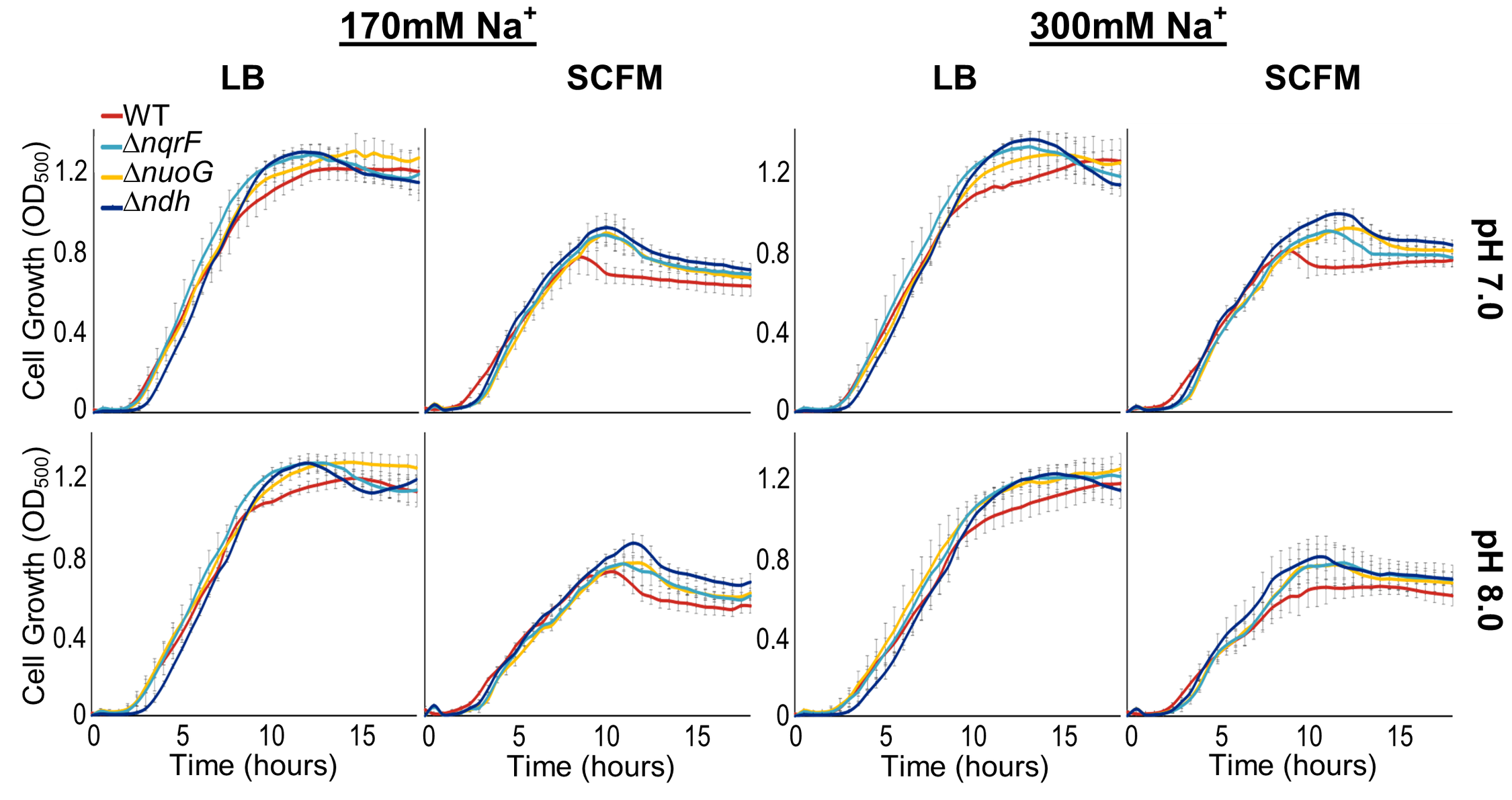

Supplement: S2 Fig — Growth curves of wild type PAO1 (red) and single deletion mutants ΔnqrF (cyan), ΔnuoG (yellow), and Δndh (dark blue) in LB and SCFM media at 170mM and 300mM NaCl concentrations, at pH 7.0 and 8.0. Changes in OD500 were measured using a Tecan Infinite M1000 Pro plate reader during 20 hours of growth at 37°C with continuous orbital shaking at 217 rpm. Each curve was constructed using two biological replicates with three technical replicates each, with standard deviation calculated accordingly and represented as error bars. (TIF) [file pone.0244142.s002.tif]

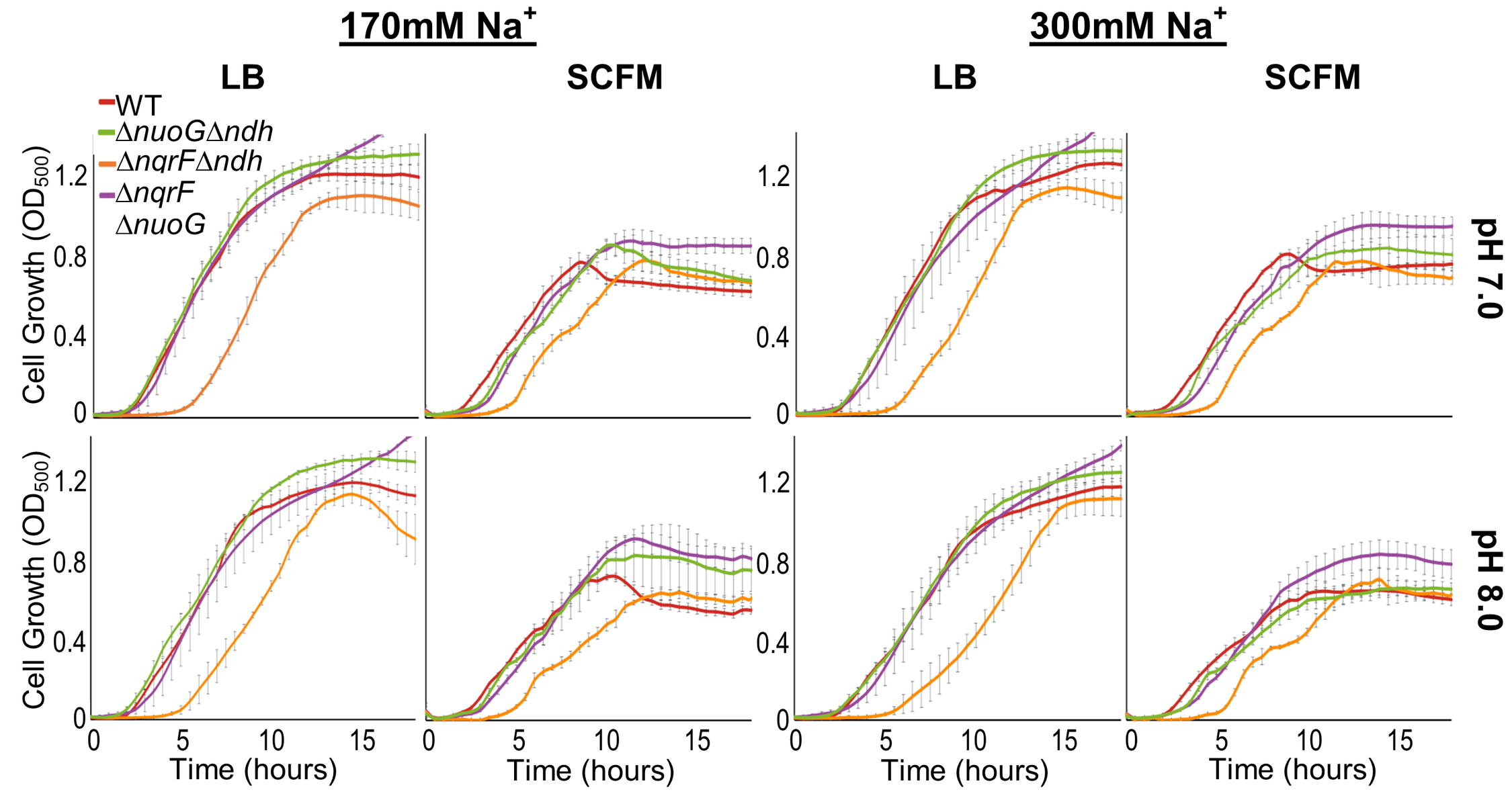

Supplement: S3 Fig — Growth curves of wild type PAO1 (red) and double deletion mutants ΔnuoGΔndh (green), ΔnqrFΔndh (orange), and ΔnqrFΔnuoG (purple) in LB and SCFM media at 170mM and 300mM NaCl concentrations, at pH 7.0 and 8.0. Changes in OD500 were measured using a Tecan Infinite M1000 Pro plate reader during 20 hours of growth at 37°C with continuous orbital shaking at 217 rpm. Each curve was constructed using two biological replicates with three technical replicates each, with standard deviation calculated accordingly and represented as error bars. (TIF) [file pone.0244142.s003.tif]

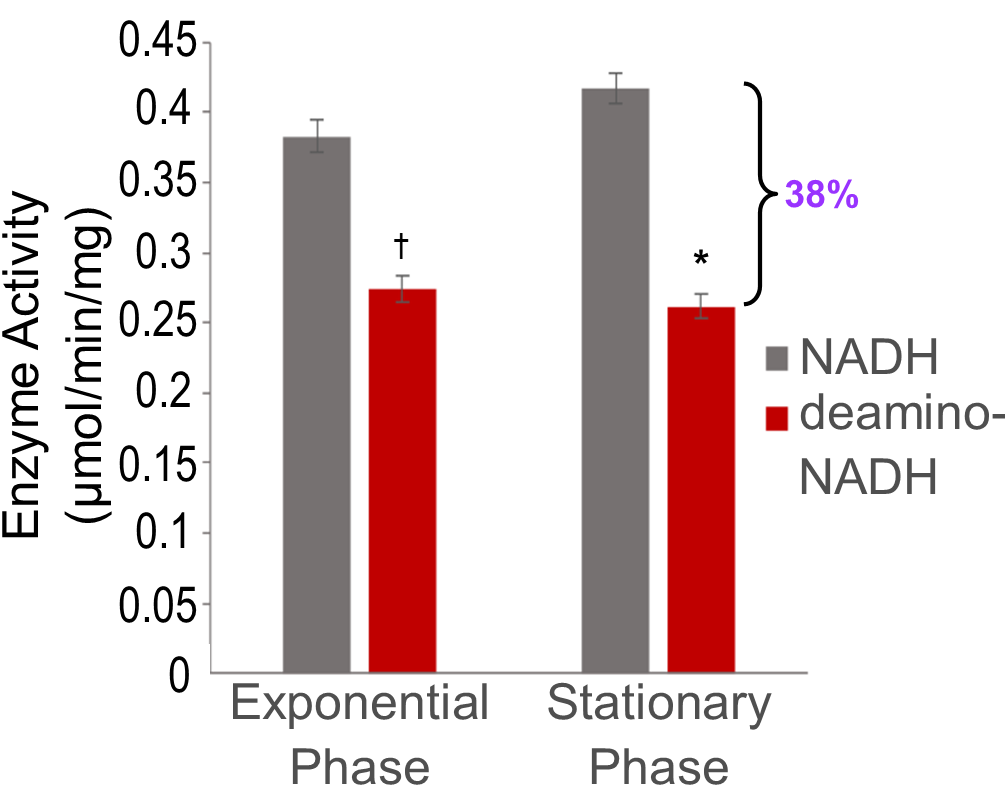

Supplement: S4 Fig — Membranes were harvested in exponential and stationary phase. Enzyme activity is defined as the μmoles of NADH (or deamino-NADH) consumed per minute per mg of membrane protein. The reaction contained: 100μM NADH, 50μM ubiquinone-1 and 100mM NaCl. Changes in absorbance were followed at 340nm (εNADH = 6.22 mM−1 cm−1). Stars indicate p-values of ≤ 0.01 compared to WT according to student’s t-test. (TIF) [file pone.0244142.s004.tif]

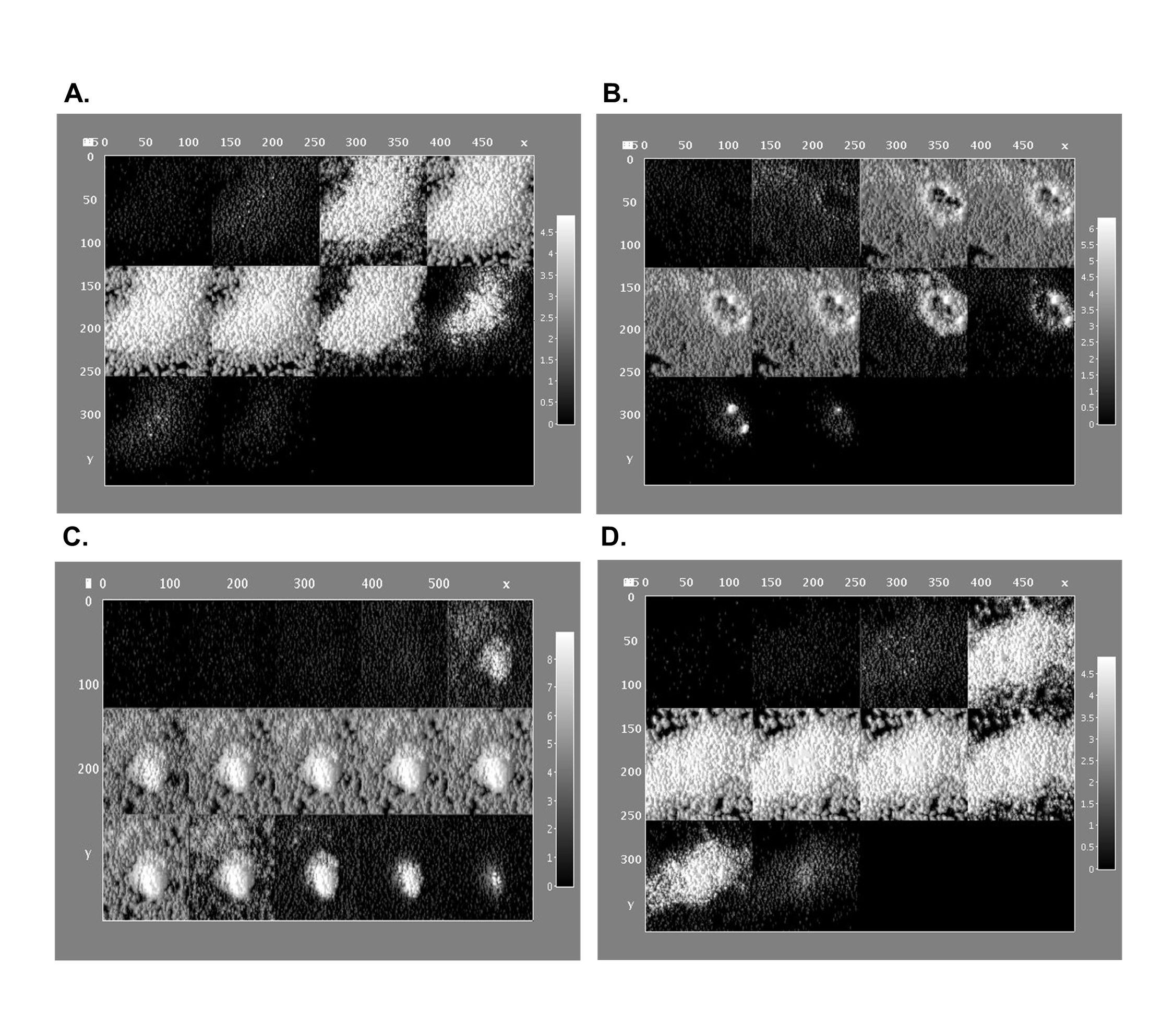

Supplement: S5 Fig — Representative surface images of biofilm produced by WT (A), Δndh (B), ΔnuoG (C), and ΔnqrF (D) at the 6-hour timepoint. (TIF) [file pone.0244142.s005.tif]

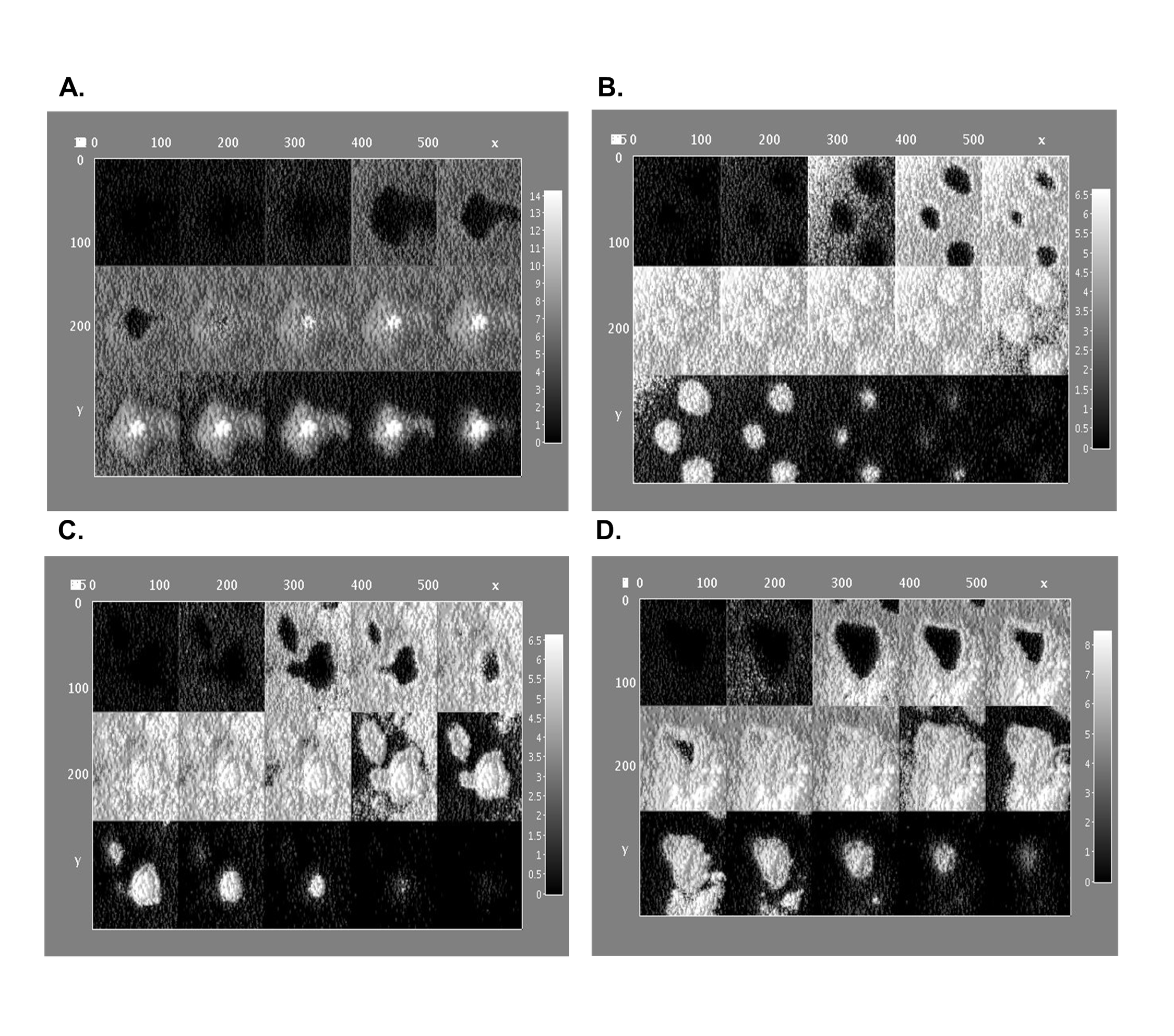

Supplement: S6 Fig — Representative surface images of biofilm produced by WT (A), Δndh (B), ΔnuoG (C), and ΔnqrF (D) at the 24-hour timepoint. (TIF) [file pone.0244142.s006.tif]

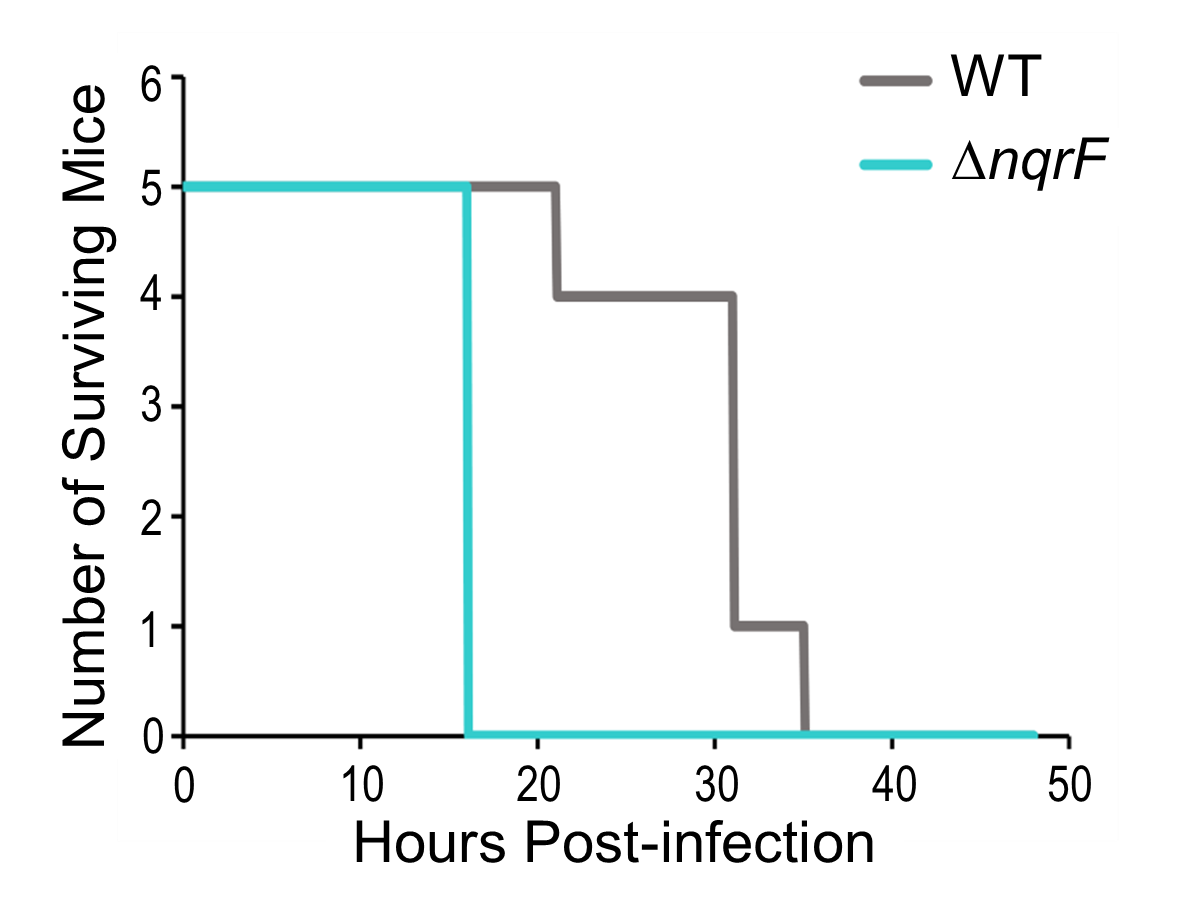

Supplement: S7 Fig — Survival of BALB/c mice after intranasal aspiration of ~4 x10^6^ CFU wild type or ΔnqrF. The survival curves were statistically different (p < 0.05; log rank test). (TIF) [file pone.0244142.s007.tif]

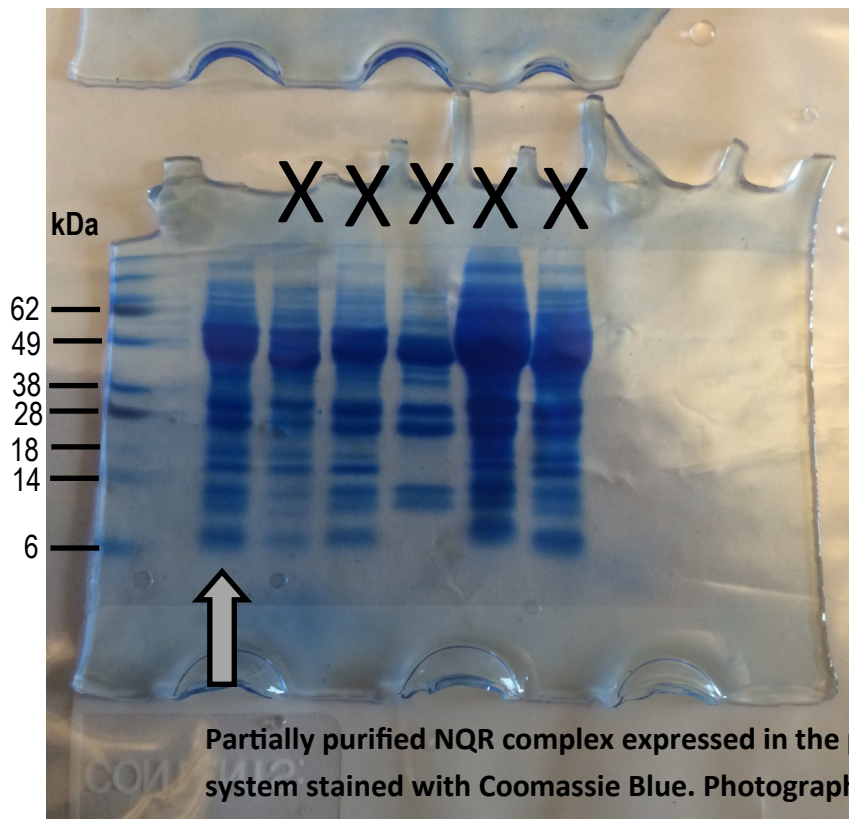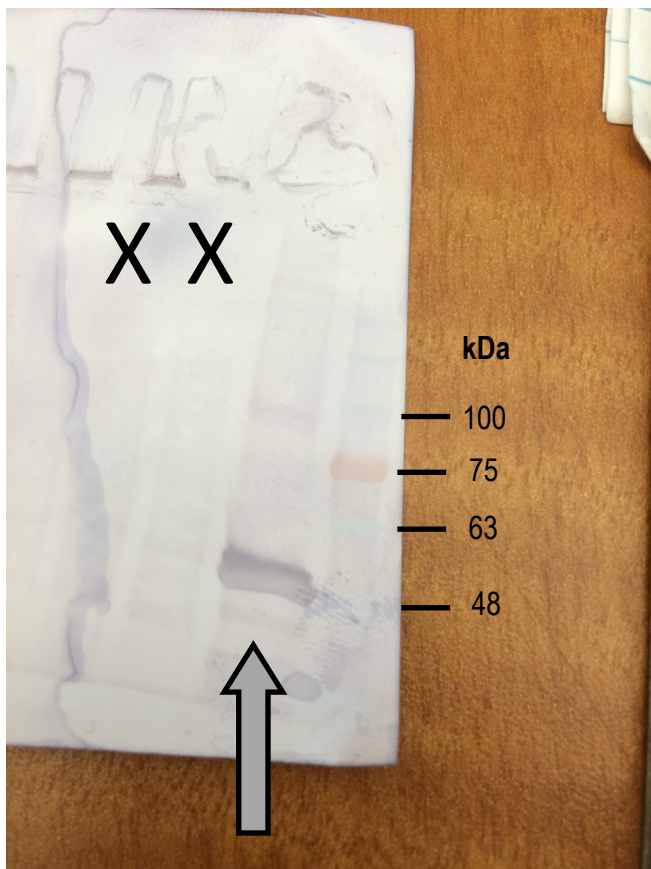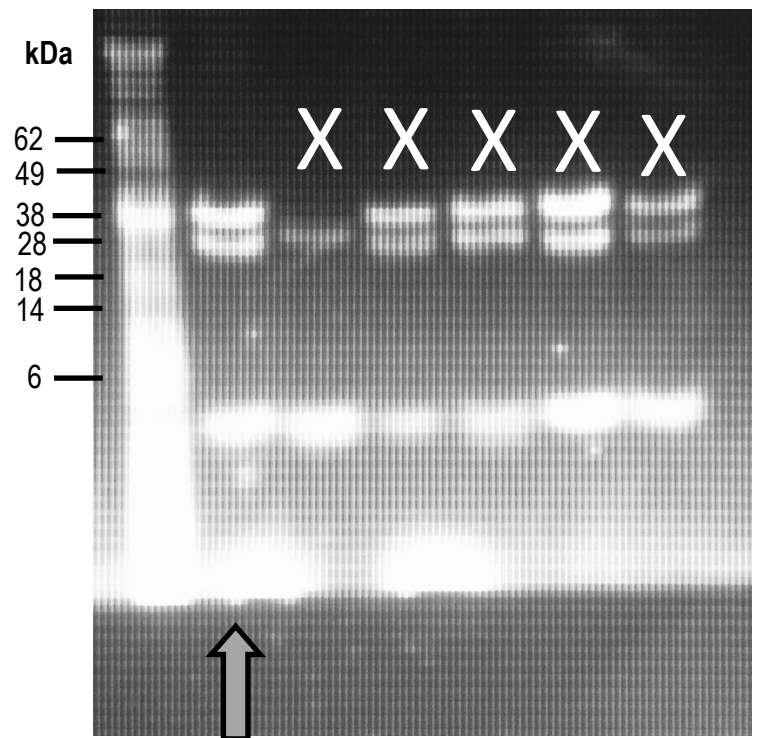

Supplement: S1 Raw images — (PDF) [file pone.0244142.s012.pdf]
