# Supplementary material for: The three NADH dehydrogenases of Pseudomonas aeruginosa: Their roles in energy metabolism and links to virulence
Source: PLoS One. 2021 Feb 3;16(2):e0244142. doi: 10.1371/journal.pone.0244142 (PMC7857637; doi:10.1371/journal.pone.0244142)
Supplement: S1 Table — a Maximum growth rates and doubling times were determined using the fit_easylinear algorithm provided in the growthrates R package [32, 33]. b Above parameters calculated from growth curves depicted in Figs 1 and 2. * indicates P-value ≤ 0.01, ** indicates P- value ≤ 0.001. (DOCX) [file pone.0244142.s008.docx]

| **Growth Parameters in LB** | | | | |
| --- | --- | --- | --- | --- |
| **Strain** | pH 7.0  170mM NaCl | pH 7.0  300mM NaCl | pH 8.0  170mM NaCl | pH 8.0  300mM NaCl |
|  | Doubling Time (min) | Doubling Time (min) | Doubling Time (min) | Doubling Time (min) |
| **PAO1** | 26.6 ± 1.8 | 22.2 ± 2.3 | 24.9 ± 1.4 | 30.6 ± 2.4 |
| **∆*ndh*** | 27.7 ± 1.6 | 26.5 ± 4.3 | 25.8 ± 1.7 | 32.8 ± 2.7 |
| **∆*nuoG*** | 30.4 ± 2.9***** | 31.6 ± 6.6***** | 33 ± 3.2***** | 41.8 ± 5.6***** |
| **∆*nqrF*** | 31.2 ± 1.1***** | 33 ± 1.5***** | 29.5 ± 2.1***** | 39 ± 2.1***** |
|  |  |  |  |  |
| **∆*nqrF*∆*nuoG*** | 39.5 ± 0.7****** | 50.5 ± 0.7****** | 44.2 ± 2.3****** | 59.1 ± 3.4****** |
| **∆*nqrF*∆*ndh*** | 44.9 ± 1.7****** | 41.5 ± 1.7****** | 39.3 ± 2.2****** | 45.8 ± 8.3****** |
| **∆*nuoG*∆*ndh*** | 34.4 ± 1.8****** | 34.8 ± 1.8****** | 32.8 ± 2.3****** | 40.4 ± 3.7***** |
