# Supplementary material for: The three NADH dehydrogenases of Pseudomonas aeruginosa: Their roles in energy metabolism and links to virulence
Source: PLoS One. 2021 Feb 3;16(2):e0244142. doi: 10.1371/journal.pone.0244142 (PMC7857637; doi:10.1371/journal.pone.0244142)
Supplement: S2 Table — a Maximum growth rates and doubling times were determined using the fit_easylinear algorithm provided in the growthrates R package [32, 33]. b Above parameters calculated from growth curves depicted in Figs 1 and 2. * indicates P-value ≤ 0.01, ** indicates P- value ≤ 0.001. (DOCX) [file pone.0244142.s009.docx]

| **Growth Parameters in SCFM** | | | | |
| --- | --- | --- | --- | --- |
| **Strain** | pH 7.0  170mM NaCl | pH 7.0  300mM NaCl | pH 8.0  170mM NaCl | pH 8.0  300mM NaCl |
|  | Doubling Time (min) | Doubling Time (min) | Doubling Time (min) | Doubling Time (min) |
| **PAO1** | 40.4 ± 3.8 | 39 ± 7.7 | 38 ± 2.4 | 32.8 ± 7.6 |
| **∆*ndh*** | 33.3 ± 1.4 | 30.9 ± 3.9***** | 27.6 ± 1.0****** | 27.2 ± 3.0***** |
| **∆*nuoG*** | 40.7 ± 3.6 | 35.9 ± 2.6 | 38 ± 1.8 | 34.4 ± 5.8 |
| **∆*nqrF*** | 37.9 ± 2.6***** | 34.9 ± 4.9 | 32.7 ± 1.8 | 29.8 ± 4.2 |
|  |  |  |  |  |
| **∆*nqrF*∆*nuoG*** | 42.1 ± 4.4 | 47.8 ± 0.8***** | 37.9 ± 2.8 | 42.8 ± 4.6***** |
| **∆*nqrF*∆*ndh*** | 42.4 ± 2.3 | 39.7 ± 8.5 | 84.6 ± 1.0****** | 79.4 ± 6.9****** |
| **∆*nuoG*∆*ndh*** | 46.7 ± 1.6***** | 40.7 ± 1.0 | 46.4 ± 2.4***** | 43.3 ± 3.3***** |
