# Supplementary material for: The three NADH dehydrogenases of Pseudomonas aeruginosa: Their roles in energy metabolism and links to virulence
Source: PLoS One. 2021 Feb 3;16(2):e0244142. doi: 10.1371/journal.pone.0244142 (PMC7857637; doi:10.1371/journal.pone.0244142)
Supplement: S3 Table — aActivities for each strain represented as a percentage of wild-type activity (100%) (DOCX) [file pone.0244142.s010.docx]

| **Strain** | **Enzymes present** | **Activity (% of WT); Exponential Phase** | **Activity (% of WT); Stationary Phase** |
| --- | --- | --- | --- |
| Wild type (PAO1) | NQR, NUO, NDH2 | 100% | 100% |
| ∆*nuoG*∆*ndh* | NQR | 48% | 48% |
| ∆*nqrF*∆*ndh* | NUO | 15% | 46% |
| ∆*nqrF*∆*nuoG* | NDH2 | 37% | 49% |
| ∆*nqrF* | NUO, NDH2 | 41% | 54% |
| ∆*nuoG* | NQR, NDH2 | 67% | 53% |
| ∆*ndh* | NQR, NUO | 62% | 62% |
| Wild type (PAO1)  w/ deamino NADH | NQR, NUO, NDH2* | 70% | 62% |
