# Supplementary material for: The three NADH dehydrogenases of Pseudomonas aeruginosa: Their roles in energy metabolism and links to virulence
Source: PLoS One. 2021 Feb 3;16(2):e0244142. doi: 10.1371/journal.pone.0244142 (PMC7857637; doi:10.1371/journal.pone.0244142)
Supplement: S4 Table — aA positive value indicates an increased expression in ΔnqrF compared to wild-type. P-values were determined using the calculations outlined in Materials and Methods. (DOCX) [file pone.0244142.s011.docx]

| **Gene** | **Function** | **Log_2_FC Exponential** | ***P*-value** | **Log_2_FC**  **Stationary** | ***P*-value** |
| --- | --- | --- | --- | --- | --- |
| **pelA** | Encode enzymes involved in Pel exopolysaccharide biosynthesis | 0.267 | 0.059 | -0.179 | 0.220 |
| **pelB** |  | 0.248 | 0.098 | 0.047 | 0.792 |
| **pelC** |  | 0.422 | 0.093 | -0.271 | 0.336 |
| **pelD** |  | 0.668 | 0.001 | -0.001 | 0.996 |
| **pelE** |  | 0.306 | 0.101 | -0.120 | 0.501 |
| **pelF** |  | 0.101 | 0.49b | -0.299 | 0.091 |
| **pelG** |  | -0.186 | 0.262 | -0.067 | 0.674 |
|  |  |  |  |  |  |
| **algD** | Encode enzymes involved in alginate biosynthesis | -0.085 | 0.630 | -0.108 | 0.476 |
| **alg8** |  | -0.026 | 0.883 | 0.015 | 0.931 |
| **alg44** |  | 0.165 | 0.373 | -0.188 | 0.392 |
| **algK** |  | 0.239 | 0.187 | 0.056 | 0.799 |
| **algE** |  | 0.408 | 0.014 | 0.010 | 0.957 |
| **algG** |  | -0.031 | 0.822 | 0.149 | 0.361 |
| **algX** |  | 0.696 | 0.001 | 0.183 | 0.395 |
| **algL** |  | 0.600 | 0.006 | 0.619 | 0.012 |
| **algI** |  | 0.124 | 0.398 | -0.066 | 0.666 |
| **algJ** |  | 0.341 | 0.052 | 0.012 | 0.962 |
| **algF** |  | 0.325 | 0.022 | -0.042 | 0.779 |
| **algA** |  | 0.417 | 0.003 | 0.005 | 0.975 |
|  |  |  |  |  |  |
| **pslA** | Encode enzymes involved in Psl exopolysaccharide biosynthesis | 1.120 | 4.90E-14 | 0.539 | 0.006 |
| **pslB** |  | 1.351 | 9.45E-13 | 0.233 | 0.159 |
| **pslC** |  | 1.231 | 1.05E-12 | 0.212 | 0.233 |
| **pslD** |  | 1.126 | 1.38E-11 | 0.132 | 0.395 |
| **pslE** |  | 1.222 | 3.64E-11 | 0.275 | 0.094 |
| **pslF** |  | 1.353 | 1.82E-12 | 0.295 | 0.070 |
| **pslG** |  | 1.150 | 6.09E-12 | 0.445 | 0.015 |
| **pslH** |  | 1.279 | 2.71E-14 | 0.435 | 0.016 |
| **pslI** |  | 1.235 | 1.23E-12 | 0.392 | 0.029 |
| **pslJ** |  | 1.155 | 1.16E-10 | 0.366 | 0.034 |
| **pslK** |  | 1.056 | 1.97E-12 | 0.299 | 0.092 |
| **pslL** |  | 1.109 | 1.88E-17 | 0.307 | 0.048 |
|  |  |  |  |  |  |
| **lasA** | LasA protease precursor | 2.725 | 8.12E-20 | -0.1253 | 0.315 |
|  |  |  |  |  |  |
| **mexE** | MexEF-OprN Multi-drug Efflux Pump | -9.012 | 0 | -5.519 | 0 |
| **mexF** |  | -8.909 | 0 | -5.063 | 0 |
| **oprN** |  | -8.404 | 0 | -4.903 | 0 |
| **PA2491** | MexS, regulator of MexEF-OprN | -3.367 | 0 | -3.851 | 0 |
|  |  |  |  |  |  |
| **mexG** | MexGHI-OpmD RND Efflux Pump | 3.254 | 4.99E-45 | -0.438 | 1.45E-05 |
| **mexH** |  | 3.220 | 1.82E-36 | -0.283 | 0.093 |
| **mexI** |  | 3.021 | 1.23E-28 | -0.506 | 0.001 |
| **opmD** |  | 3.709 | 0 | -1.113 | 1.29E-27 |
