# Supplementary material for: The three NADH dehydrogenases of Pseudomonas aeruginosa: Their roles in energy metabolism and links to virulence
Source: PLoS One. 2021 Feb 3;16(2):e0244142. doi: 10.1371/journal.pone.0244142 (PMC7857637; doi:10.1371/journal.pone.0244142)
Supplement: Attachment — Submitted filename: Reviewers commments and response 12.2.20.docx [file pone.0244142.s013.docx]

**Response to Reviewers:**

We have included our response to the Reviewers’ comments below. Our responses are writing below each comment in blue.

The work is clear, solid and of interest. A concerned was raised by Reviewer #1 about your mice infection data as the results of just one experiment are shown in Fig.8A, although the experiment was repeated twice. Please address this concern, provide the requested details about the mutant strains and revise the manuscript as constructively advised by both reviewers.

See response to Reviewer 1 below.

~~1. Please ensure that your manuscript meets PLOS ONE's style requirements, including those for file naming. The PLOS ONE style templates can be found at~~

<https://journals.plos.org/plosone/s/file?id=wjVg/PLOSOne_formatting_sample_main_body.pdf> and

<https://journals.plos.org/plosone/s/file?id=ba62/PLOSOne_formatting_sample_title_authors_affiliations.pdf>

2. PLOS ONE now requires that authors provide the original uncropped and unadjusted image underlying all blot or gel results reported in a submission’s figure or Supporting Informatin files.

This policy and the journal’s other requirements for blot/gel reporting and figure preparation are described in detail at <https://journals.plos.org/plosone/s/figures#loc-blot-and-gel-reporting-requirements> and <https://journals.plos.org/plosone/s/figures#loc-preparing-figures-from-image-files>. When you submit your revised manuscript, please ensure that your figures adhere fully to these guidelines and provide the original underlying images for all blot or gel data reported in your submission. See the following link for instructions on providing the original image data: <https://journals.plos.org/plosone/s/figures#loc-original-images-for-blots-and-gels>.

In your cover letter, please note whether your blot/gel image data are in Supporting Information or posted at a public data repository, provide the repository URL if relevant, and provide specific details as to which raw blot/gel images, if any, are not available. Email us at plosone@plos.org if you have any questions.

The original pictures of gels and blots are now included in the Supporting Information file.

3. PLOS requires an ORCID iD for the corresponding author in Editorial Manager on papers submitted after December 6th, 2016. Please ensure that you have an ORCID iD and that it is validated in Editorial Manager. To do this, go to ‘Update my Information’ (in the upper left-hand corner of the main menu), and click on the Fetch/Validate link next to the ORCID field. This will take you to the ORCID site and allow you to create a new iD or authenticate a pre-existing iD in Editorial Manager. Please see the following video for instructions on linking an ORCID iD to your Editorial Manager account: <https://www.youtube.com/watch?v=_xcclfuvtxQ>

4. We note that you have included the phrase “data not shown” in your manuscript. Unfortunately, this does not meet our data sharing requirements. PLOS does not permit references to inaccessible data. We require that authors provide all relevant data within the paper, Supporting Information files, or in an acceptable, public repository. Please add a citation to support this phrase or upload the data that corresponds with these findings to a stable repository (such as Figshare or Dryad) and provide and URLs, DOIs, or accession numbers that may be used to access these data. Or, if the data are not a core part of the research being presented in your study, we ask that you remove the phrase that refers to these data.

The phrase “data not shown” (Line 376) was eliminated from the manuscript.

5. Please include captions for your Supporting Information files at the end of your manuscript, and update any in-text citations to match accordingly. Please see our Supporting Information guidelines for more information: http://journals.plos.org/plosone/s/supporting-information.

[Note: HTML markup is below. Please do not edit.]

**Reviewers' comments:**

Reviewer's Responses to Questions

**Comments to the Author**

1. Is the manuscript technically sound, and do the data support the conclusions?

The manuscript must describe a technically sound piece of scientific research with data that supports the conclusions. Experiments must have been conducted rigorously, with appropriate controls, replication, and sample sizes. The conclusions must be drawn appropriately based on the data presented.

Reviewer #1: Partly

Reviewer #2: Yes

2. Has the statistical analysis been performed appropriately and rigorously?

Reviewer #1: Yes

Reviewer #2: Yes

3. Have the authors made all data underlying the findings in their manuscript fully available?

The [PLOS Data policy](http://www.plosone.org/static/policies.action#sharing) requires authors to make all data underlying the findings described in their manuscript fully available without restriction, with rare exception (please refer to the Data Availability Statement in the manuscript PDF file). The data should be provided as part of the manuscript or its supporting information, or deposited to a public repository. For example, in addition to summary statistics, the data points behind means, medians and variance measures should be available. If there are restrictions on publicly sharing data—e.g. participant privacy or use of data from a third party—those must be specified.

Reviewer #1: Yes

Reviewer #2: Yes

4. Is the manuscript presented in an intelligible fashion and written in standard English?

PLOS ONE does not copyedit accepted manuscripts, so the language in submitted articles must be clear, correct, and unambiguous. Any typographical or grammatical errors should be corrected at revision, so please note any specific errors here.

Reviewer #1: Yes

Reviewer #2: Yes

5. Review Comments to the Author

Please use the space provided to explain your answers to the questions above. You may also include additional comments for the author, including concerns about dual publication, research ethics, or publication ethics. (Please upload your review as an attachment if it exceeds 20,000 characters)

Reviewer #1: This manuscript describes the phenotypes of deletion strains for NDH-2, NQR and NUO in P. aeruginosa. The manuscript is well written and clearly describes differences between the reported results and existing literature. The use of LB and SCFM for experimentation, as well as stationary and log phase is helpful. The authors have used PCR to confirm the identities of the transposon strains used.

The finding that MexEF-OprN is differentially regulated in the DNqr strain is interesting.

The finding that

Major Comments

1. Please show in the manuscript (or provide to the reviewers) the data for the other replicate of the mouse experiment. Demonstration of only one replicate is not adequate for evaluation.

We have included data for the replica of the mouse experiments (Figure 8). These data are included in S7 Figure.

Minor Comments

• Line 63, would briefly mention the ability of P. aeruginosa to anaerobically respire. This adds even more branches to the respiratory chain (and is an acceptable limitation to this data set).

We have modified the text to include the referee’s comment.

• Format organism names in the bibliography uniformly.

This was corrected.

• Table 1: Refer to insertion deletion mutants using ::Tn or something similar to differentiate the from the chromosomal deletions (see <https://www.gs.washington.edu/labs/manoil/libraryindex.htm>). Are the double mutants done in the ::Tn backgrounds? If so, was the abx cassette from a Tn left in place? For the purposes of the body of the manuscript, the chosen nomenclature is adequate because it impr9ves clarify of the writing.

We have now included a clarification of  the nomenclature used for the mutants (Line 11.) The transposon mutants were also described in Table 1 using the “ ::Tn nomenclature”.

• For growth curves, please present data as log-OD (Figures 1, 2, S1, etc).

We have included new Figures 1 and 2 presenting the growth curves in log scale. The original figures are included as S2 and S3 Figures.

• Table 3: Doublecheck the > and < signs in the  Dndh column

This was corrected.

• Line 541 and 542: comment still present in document from Word.

This was corrected.

**Reviewer #2**: Hreha et al. characterize the function of the three NADH dehydrogenases in P. aeruginosa. They determine that NQR has the greatest dehydrogenase activity and that its absence increases the cytotoxin pyocyanin, macrophage death, and mouse mortality. Their results suggest a model in which NADH dehydrogenase activity through NQR negatively regulates pyocyanin and virulence. Overall, the topic is very interesting and the results provide further evidence for a causal link between NADH metabolism and pathogenesis. My only comments are requests for further clarity on the issues below.

Minor issues:

1. Line 308: ref 29 does not appear to be an appropriate reference for SCFM medium

This was corrected.

2. Fig. 7. The y-axis appears to be the fold-change in macrophage death compared to the bacteria-free control. However, the extent of death in bacteria-free control should be the minimal and the death in the other groups is therefore difficult to interpret. I suggest the authors plot something more intuitive such as % cell death or % viability instead.

We have included a new Figure 7. This figure shows the % of cell death.

3. Section starting at line 656: Title mentions biofilm formation but this is not discussed in the paragraph that follows.

The title has been modified.

4. Line 229: “4V%” looks like a typo.

This was corrected.

5. Lines 446 & 447: “propodium” should be “propidium”

This was corrected.

6. Line 473: There are no stars in Fig. 5.

This was corrected.

6. PLOS authors have the option to publish the peer review history of their article ([what does this mean?](https://journals.plos.org/plosone/s/editorial-and-peer-review-process#loc-peer-review-history)). If published, this will include your full peer review and any attached files.

If you choose “no”, your identity will remain anonymous but your review may still be made public.

**Do you want your identity to be public for this peer review?** For information about this choice, including consent withdrawal, please see our [Privacy Policy](https://www.plos.org/privacy-policy).

Reviewer #1: No

Reviewer #2: No

[NOTE: If reviewer comments were submitted as an attachment file, they will be attached to this email and accessible via the submission site. Please log into your account, locate the manuscript record, and check for the action link "View Attachments". If this link does not appear, there are no attachment files.]

While revising your submission, please upload your figure files to the Preflight Analysis and Conversion Engine (PACE) digital diagnostic tool, <https://pacev2.apexcovantage.com/>. PACE helps ensure that figures meet PLOS requirements. To use PACE, you must first register as a user. Registration is free. Then, login and navigate to the UPLOAD tab, where you will find detailed instructions on how to use the tool. If you encounter any issues or have any questions when using PACE, please email PLOS at figures@plos.org. Please note that Supporting Information files do not need this step.

*In compliance with data protection regulations, you may request that we remove your personal registration details at any time.* [*(Remove my information/details)*](https://www.editorialmanager.com/pone/login.asp?a=r)*. Please contact the publication office if you have any questions.*
